# Turmeric extract (*Curcuma longa* L.) regulates hepatic toxicity in a single ethanol binge rat model

**DOI:** 10.1016/j.heliyon.2022.e10737

**Published:** 2022-09-22

**Authors:** Hwa-Young Lee, Geum-Hwa Lee, The-Hiep Hoang, Seung Wook Kim, Choon Gil Kang, Jae Hyeok Jo, Myoung Ja Chung, Kyunghyun Min, Han-Jung Chae

**Affiliations:** a Research Institute of Clinical Medicine of Jeonbuk National University-Biomedical Research Institute & Non-Clinical Evaluation Center, Jeonbuk National University Hospital, Jeonju, Jeonbuk 54907, South Korea; b Ottogi Research Center, Anyang, Gyeonggi-do, 14060, South Korea; c Department of Pathology, Jeonbuk National University Medical School, Jeonju, Jeonbuk 54907, South Korea; d School of Pharmacy and Pharmaceutical Research Institute of Korea Unification, Jeonbuk National University, Jeonju, Jeonbuk 54896, South Korea

**Keywords:** Alcohol, Binge drinking, Turmeric extract, Oxidative stress, Liver damage

## Abstract

Hepatic alcohol clearance is a key factor to overcome alcohol hangovers, and over the period, alcohol hangovers may lead to inflammation and oxidative stress. Natural food products with high antioxidant and anti-inflammatory effects might contribute to hepatic alcohol clearance, a hypothesis in this study. The present study aimed to evaluate the influence of turmeric (*Curcuma longa L., Zingiberaceae*) is an herbal product having antioxidant and anti-inflammatory activities, on alcohol metabolism using binge alcohol drinking rat model. In vivo investigations revealed that pretreatment with turmeric extract enhanced alcohol dehydrogenase (ADH) and aldehyde dehydrogenase (ALDH) activities upon binge ethanol (3 g/kg). Additionally, pretreatment with turmeric extract regulated CYP2E1 activity and levels of reactive oxygen species (ROS), Bax, Bcl-2, and inflammatory mediators like IL-1β, IL-6, and TNF-α. Moreover, turmeric extract upregulated superoxide dismutase, catalase, and glutathione peroxidase activities in liver tissues. Together, these observations shed light on the potential beneficial effects of turmeric extract against acute liver toxicity. The results offer an alternative natural functional food product, turmeric extract, to prevent the negative implications of binge drinking.

## 1 Introduction

Consumption of alcohol is an age-old means of socializing and stress relief. However, modernization changed the way alcohol is consumed. Societies in the modern era have developed a binge drinking culture and adopted frequent alcohol intake, raising health and social problems. Health issues like hangovers and severe liver damage are common among people with the habit of binge drinking. The liver is the most vulnerable organ to alcohol consumption. However, it has the exceptional regenerative ability and can handle toxic byproducts of alcohol well. Nonetheless, acute and chronic alcohol consumption potentially damages the liver cells and negatively affects its regenerative capacity. However, liver exhibits the exceptional regenerative ability to handle toxic byproducts of alcohol. Notwithstanding, acute and chronic alcohol consumption leads to abnormal biochemical and inflammatory changes, potentially damaging the liver cells and negatively affecting its regenerative capacity.

In general, alcohol hangovers are symptomatic. The most common symptoms are headache, thirst, dry mouth, drowsiness, vomiting, and fatigue (Penning et al., 2012; Swift and Davidson, 1998). However, the severity of these symptoms varies with the type and volume of alcohol consumed and varies from person to person (Hogewoning et al., 2016; Verster et al., 2018). Alcohol metabolism begins with the formation of acetaldehyde in the presence of alcohol dehydrogenase (ADH) and cytochrome P450E1 (CYP2E1) (Jiang et al., 2020). Further, aldehyde dehydrogenase (ALDH) converts acetaldehyde into acetate, which triggers multiple hangover symptoms via the formation of free radicals, enhanced inflammatory mediators, and an imbalance in neurotransmitters. Collectively, these factors influence the etiology of hangovers and liver dysfunction (Palmer et al., 2019; Verster et al., 2020). However, substances triggering ethanol metabolism can alleviate hangovers and eventually lowers liver damage. Currently, several products are available that can reduce hangover symptoms. Many of these products contain natural ingredients, such as vegetables, herbs, and fruits. These products potentially remove alcohol metabolites, suppress inflammatory mediators, and promote antioxidant enzyme activities (Palmer et al., 2019). However, these products can relieve only selected symptoms depending on the ingredients used (Jayawardena et al., 2017), and several products require double-blind clinical trials to confirm their potential positive effects on hangover symptoms. Moreover, the efficacy of a product depends on an individual's health (Pittler and Ernst, 2003). Turmeric (*Curcuma longa* L.), a perennial herb in the ginger family, is frequently used as a household remedy for various diseases. Curcumin is a chemical produced by turmeric extract. Several in vitro and in vivo investigations have demonstrated the broad therapeutic actions of curcumin, such as anti-inflammatory, antioxidant, antifibrotic, and anti-tumor effects (Aggarwal, 2010; Epstein et al., 2010). Additionally, curcumin administration protects the liver against iron overdose, ethanol, cholestasis, and thioacetamide (Rivera-Espinoza and Muriel, 2009). However, the mechanism by which curcumin exerts its effects remains unclear. In this study, the effect of turmeric extract on alcohol metabolism was assessed by monitoring inflammatory mediators, oxidative stress signals, and enzymes involved in alcohol metabolism in a single ethanol binge rodent model.

## 2 Materials and methods

### 2.1 Preparation of turmeric extract

Turmeric extract was obtained from Ottogi Co (Anyang, Gyeonggi-do, South Korea). Dried turmeric rhizomes purchased from India were ground, and weighed, then extracted in 50% ethanol. The mixture was filtered, and the resulting liquid was concentrated to yield a dark yellow extract. Finally, the concentrated extract was spray dried to make powder and stored at -20 °C until use.

### 2.2 Chemicals

All enzyme analyses were performed using commercial kits (BioVision, Milpitas, CA, USA). Aspartate aminotransferase (AST) and alanine aminotransferase (ALT) levels were determined using commercially available kits (Asan Pharma, Hwasung, Gyeonggi-do, South Korea). Thiobarbituric acid (TBA) and silymarin were supplied by Sigma-Aldrich (St Louis, MO, USA). The chemicals and reagents utilized in this study were of the highest quality.

### 2.3 Animals and experimental design

Male SD rats aged seven weeks were obtained from Central Lab Animal Inc. (Seoul, South Korea). Rats were looked after and maintained under typical living conditions, which included a 12 h light-dark cycle with sufficient food and water. All the animals were fed a normal chow diet. All animal tests were conducted in compliance with the regulations specified by the Jeonbuk National University Hospital Animal Care and Use Committee (CUH-IACUC-2019-12). Animals were acclimatized for approximately a week, then randomly separated into five groups. Each group consisted of eight animals. Experimental groups in the study are as follows: control (saline); EtOH (ethanol 3 g/kg rats); Turmeric extract 100 (ethanol 3 g/kg + turmeric extract 100 mg/kg mice); turmeric extract 200 (ethanol 3 g/kg + turmeric extract 200 mg/kg rats); and turmeric extract 300 (ethanol 3 g/kg + turmeric extract 300 mg/kg rats). All the solutions were administered 0.5 h before ethanol administration via oral gavage. Tissue and whole blood samples were obtained before sacrificing the animals. For analysis, whole blood was collected at a specified time (0, 0.5, 1, 3, and 5 h) after ethanol administration. The obtained liver tissue was homogenized in a buffer containing 250 mM sucrose, 50 mM Tris-HCl (pH 7.4), and 1 mM EDTA, then refrigerated at -80 °C or in 4% paraformaldehyde.

### 2.4 Evaluation of ethanol and acetaldehyde levels

Blood alcohol levels were determined using an ethanol quantification assay kit (Megazyme International, Ireland) per the manufacturer’s instructions. Briefly, 50 μL of serum was mixed with distilled water (1 mL), 0.02% sodium azide (100 μL), NAD + reagent (100 μL), and ALDH solution (25 μL). The absorbance (A1) was measured at 340 nm after 2-min incubation at room temperature. Then, the reaction mixture was incubated for 5 min at room temperature with 10 μL ADH. The absorbance (A2) was measured at 340 nm. The blood alcohol levels were calculated based on the change in absorbance (A2-A1).

### 2.5 Determination of alcohol dehydrogenase (ADH) activity

ADH activities in the liver and serum were measured using an ADH activity assay Kit (K787, *BioVision*) according to the manufacturer’s protocol. Briefly collected supernatant from liver homogenates and whole blood were diluted at 1:10 with ADH assay buffer. The reaction mixture was prepared by adding 82 μL ADH assay buffer, 8 μL developer, and 10 μL isopropanol. A standard curve was prepared in 0, 2, 4, 6, 8, and 10 nmol per well by diluting 1 mM NADH standard. About 50 μL of reaction mixture was added onto standard and sample wells and incubated at room temperature for 3 min. The absorbance was measured every 5 min for 120 min at 450 nm.

### 2.6 Determination of NAD-dependent aldehyde dehydrogenase (ALDH) activity

Serum ALDH activity was measured using an ALDH activity colorimetric assay Kit (K731-100, BioVision). Briefly, a 1:10 buffer diluted sample was mixed with reaction solution containing ALDH assay buffer, substrate mix, and acetaldehyde. The reaction mixture (50 μL) was incubated at room temperature for 5 min, and the absorbance at 450 nm was measured every 5 min for up to 60 min. To calculate ALDH activity (mU/mL), the amount of NADH released was divided by the product of the reaction time and sample volume. The standard curve at 0, 2, 4, 6, 8, and 10 nmol were prepared by diluting 1 mM NADH standard.

### 2.7 Measurement of antioxidant enzyme activities in the liver

Liver homogenates were used to estimate the activities of catalase (CAT; #K773), superoxide dismutase (SOD; #K335), and glutathione peroxidase (GPx; #K762) using commercial kits from BioVision. All the measurements were carried out in accordance with the manufacturer’s recommendations.

### 2.8 Estimation of CYP2E1 activity

CYP2E1 activity was calculated as described previously (Siregar et al., 2020). Briefly, homogenized liver tissue was centrifuged to isolate microsomal pellets and suspended in 0.15 M KCl. The protein levels in microsomal lysates were measured using a bicinchoninic acid protein assay kit (23225, Thermo Fisher Scientific, Waltham, MA, USA). About 100 μg of microsomal lysate was mixed with the reaction buffer and incubated at 37 °C for 1 h. Trichloroacetic acid (20%) was used to stop the reaction, and 10 N NaOH was added. Finally, the absorbance was determined at 510 nm with a microplate reader (VERSAmax™, Molecular Devices, California, USA). CYP2E1 activity was calculated by multiplying the sample absorbance using an extinction coefficient of 9.53 × 10^5^/M/cm.

### 2.9 Measurement of reactive oxygen species (ROS) using dihydroethidium (DHE)

ROS levels were detected using DHE. Fixed tissues were exposed to 10 mM DHE at 37 °C for 30 min. Then, the exposed tissues were washed with 3x PBS and imaged using a confocal microscope. Confocal microscopy was used to acquire the images. Relative fluorescence intensity was determined with ImageJ (NIH, Bethesda, MD, USA).

### 2.10 Reverse transcription-PCR and real-time PCR

Total RNA was isolated with TRIzol reagent (Thermo Fisher Scientific) per the manufacturer’s recommendations. 1 μg of total RNA was reverse transcribed with the iScript™ cDNA synthesis kit (Bio-Rad, California, USA) and quantified using SYBR Green Supermix (Bio-Rad). RT-PCR and qPCR were performed as described earlier (Yang et al., 2019).

### 2.11 Immunoblotting

Immunoblotting was conducted as reported earlier with minor changes (Lee et al., 2020). The protein samples (20-40 μg) were separated and transferred onto a PVDF membrane. Then, specific antibodies were incubated with blocked membranes. The following antibodies were used in the study. Anti-Bax (1:200 dilution; Santa Cruz Biotechnology, USA), anti-cytochrome C (1:1000), anti-cleaved caspase-3 (1:1000), anti-VDAC (1:1000) from Cell Signaling, USA. Anti-β-actin antibody (1:5000; Thermo Fisher Scientific). All the signals were detected and visualized with the ECL detection method.

### 2.12 Hematoxylin and eosin (H&E) staining

Liver tissues were fixed using 10% (v/v) buffered formalin, embedded in paraffin, and cut into 5 μm thick sections. These sections were deparaffinized, rehydrated, and stained with hematoxylin and eosin stain. Stained sections were observed under an Olympus DP70 microscope (Olympus Optical Co., Tokyo, Japan) and analyzed using ImageJ software (NIH, USA).

### 2.13 Statistical analysis

All statistical analyses were performed using the GraphPad Prism 5.01 software. One-way ANOVA followed by Tukey’s post-hoc test was performed for multiple comparisons. The *p*-value was set at < 0.05. Data were expressed as mean ± SEM.

## 3 Results

### 3.1 Influence of turmeric extract on ethanol metabolism

**Figure 1:**
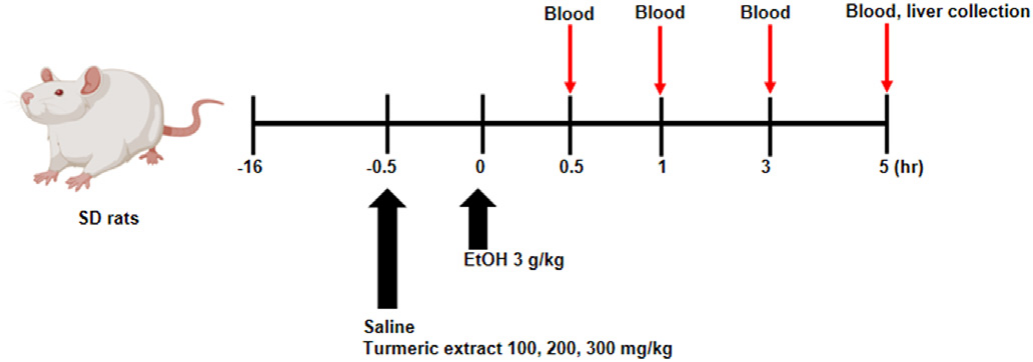
Schematic representation showing treatment time frame and sample collection time points. At the beginning of the experiment (Day 0), rats received a single high dose of ethanol. The treatment dose was estimated based on the initial weights of the experimental animals. Saline and turmeric *extracts (100, 200,* 300 mg/kg*)* were pre-treated 30 min before ethanol treatment. All the solutions were given via oral gavage. EtOH; ethanol.

**Figure 2:**
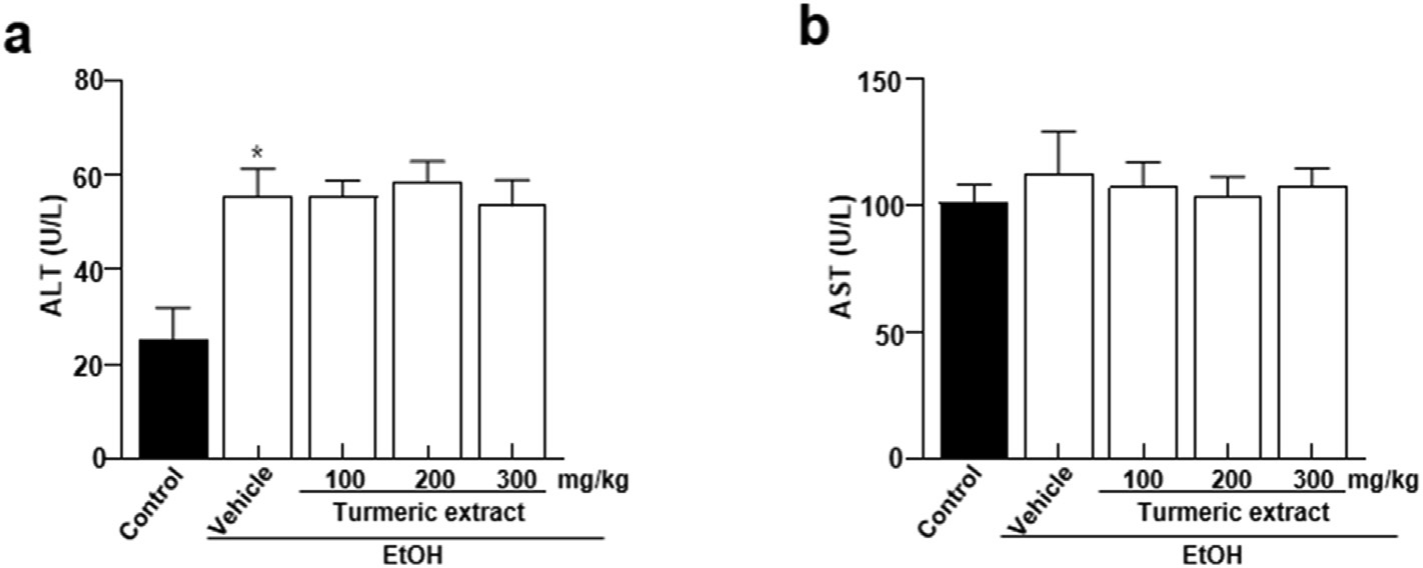
Effect of turmeric extract on the activity of hepatic enzymes in alcohol-induced SD rats. (a) ALT and (b) AST were assessed 5 h after ethanol bingeing. Data are shown as mean ± SEM (n = 8). *p* < 0.05 (#) represent differences from the ethanol-treated (EtOH) group. ALT, alanine transaminase; AST, aspartate transaminase; EtOH; ethanol.

**Figure 3:**
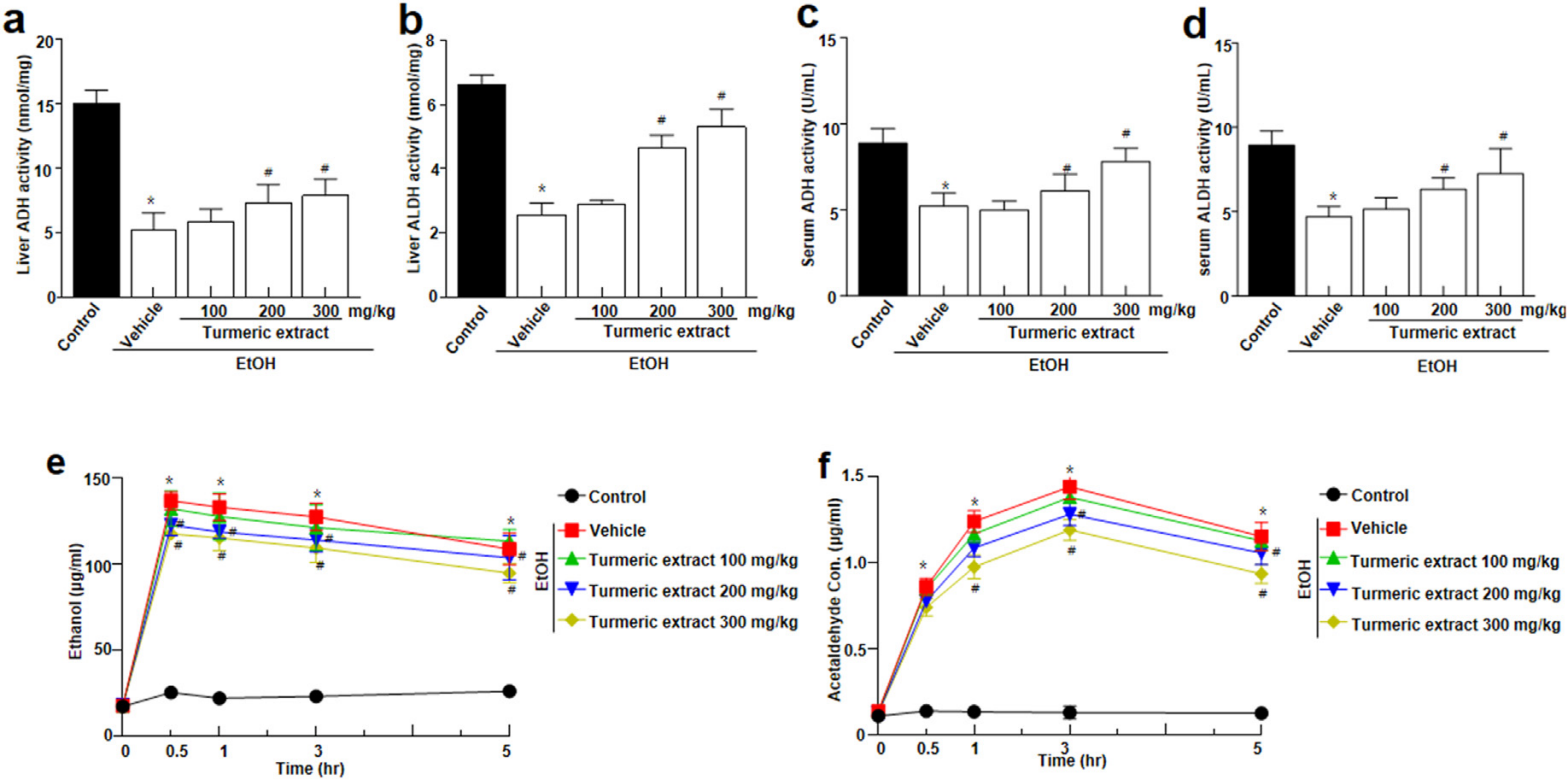
Effect of turmeric extract on alcohol metabolism in alcohol-induced SD rats. (a–d) ADH and ALDH activities were assessed after administering a single high dose (3 g/kg) of ethanol. (e) Ethanol and (f) acetaldehyde levels were evaluated at a predetermined time (0, 0.5, 1, 3, and 5 h) after administering a single high dose (3 g/kg) of ethanol. Data are expressed as mean ± SEM (n = 8). *p* < 0.05(#) represent differences from the EtOH treated group. ADH, alcohol dehydrogenase; ALDH, aldehyde dehydrogenase; EtOH; ethanol.

To determine the beneficial effects of turmeric extract on alcohol toxicity *in vivo*, rats were administered 100, 200, and 300 mg/kg of turmeric extract prior to bingeing with 3 g/kg ethanol. The experimental design of this study is illustrated in Figure 1. Serum ALT and AST are standard parameters used to assess hepatocyte injury (Tang et al., 2017). The ethanol group showed a significant difference in ALT level. However, AST levels remained unchanged compared to the ethanol group (Figure 2a-b). Next, alcohol-metabolizing enzymes like ADH and ALDH were measured in the liver and serum. Moreover, increased levels of ADH and ALDH were observed in the turmeric extract-treated group compared to that in the ethanol-treated group (Figure 3a-d). It is a well-known fact that ethanol concentrations in the body influence its metabolism. Thus, ethanol and acetaldehyde levels in blood were recorded at 0, 0.5, 1, 3, and 5 h after bingeing with ethanol. Ethanol and acetaldehyde concentration in the blood was enhanced significantly at 0.5 h compared to 0 h and then decreased with time in all the experimental groups (Figure 3e-f). However, the administration of turmeric extract effectively reduced the ethanol and acetaldehyde levels in the ethanol group, indicating the turmeric extract expedited alcohol metabolism in the animal model.

### 3.2 Influence of turmeric extract on oxidative stress

**Figure 4:**
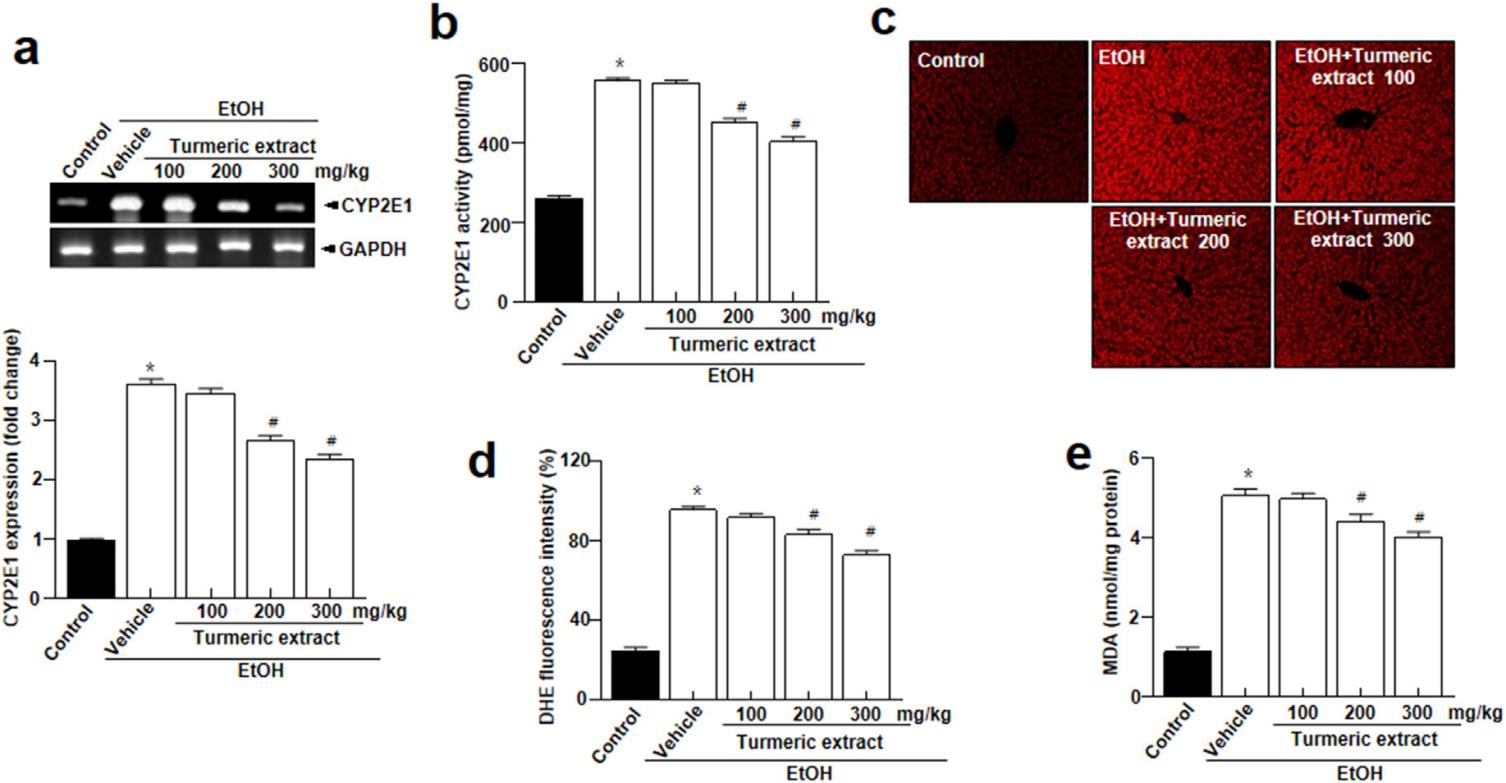
Turmeric extract suppressed ethanol-induced CYP2E1 activity and oxidative stress. (a) CYP2E1 mRNA expression in the liver tissues (upper). Quantitative analysis of mRNA expression (bottom). (b) CYP2E1 activity in the liver lysates. (c) Representative dihydroethidium (DHE)-stained images depicting ROS production and (d) its quantification in each group. Scale bars, 20 μm. (e) Levels of MDA in each experimental group. Data represent mean ± SEM (n = 8). ∗*p* < 0.05 vs control, #*p* < 0.05 vs the ethanol-treated (EtOH) group. Full and non-adjusted images of immunoblots were shown in the supplementary material (Supplementary Figure 1). EtOH, ethanol.

Acetaldehyde metabolism involves CYP2E1, a highly expressed membrane protein in the liver (Hyun et al., 2021). ROS are produced when CYP2E1 utilizes oxygen for alcohol metabolism, leading to tissue damage and oxidative stress (Jiang et al., 2020). In this study, CYP2E1 mRNA expression levels were highly enhanced in the EtOH group (Figure 4a). However, turmeric extract*-*treated groups had significantly lower CYP2E1 mRNA expression levels. Similar results were obtained for CYP2E1 activity (Figure 4b). Furthermore, turmeric extract-treated groups showed significantly lower ROS production than the EtOH group (Figure 4c-d). Additionally, malondialdehyde (MDA), an indicator of lipid peroxidation reflecting the intensity of free radical attack on hepatocytes, was significantly enhanced in the EtOH group (Gawel et al., 2004), was significantly enhanced in the EtOH group (Figure 4e). However, the MDA levels showed a decreasing trend with the increasing dose of turmeric extract. These results indicate that turmeric extract could protect the liver from oxidative stress caused by acute alcohol use.

### 3.3 Influence of turmeric extract on the activity of antioxidant enzymes

**Figure 5:**
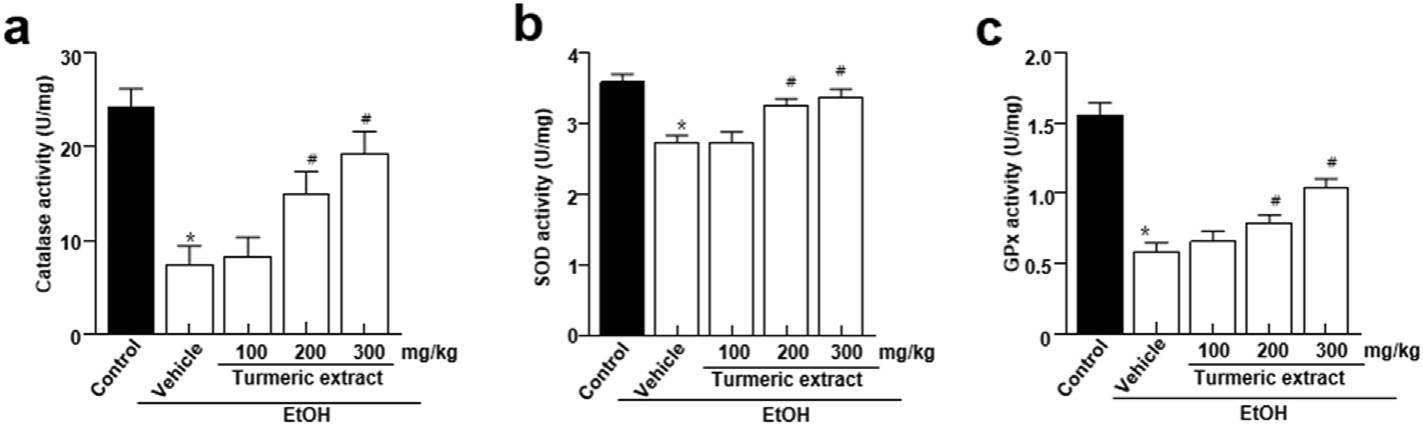
Influence of turmeric extract on antioxidant enzyme activities in alcohol-induced SD rats. (a–c) Activities of catalase, SOD, and GPx in the liver tissue. Data are expressed as mean ± SEM (n = 8). p < 0.05 (#) represent differences from the ethanol-treated (EtOH) group. EtOH, ethanol; SOD, Superoxide dismutase; GPx, Glutathione peroxidases.

Oxidative stress is strongly linked to ethanol-induced liver injury and alcoholic liver disease pathogenesis (Dey and Cederbaum, 2006). The effect of turmeric extract on the antioxidant status was determined by measuring the activity of antioxidant enzymes such as catalase, superoxide dismutase (SOD), and glutathione peroxydase (GPx). Liver tissues from the EtOH group showed considerably lower catalase activity than the vehicle group. However, pre-administration of turmeric extract recovered the decreased catalase activity in a dose-dependent manner (Figure 5a). Furthermore, SOD and GPx activities were significantly lower in the EtOH group (Figure 5b-c). Pre-administration of turmeric extract recovered the decreased activities of these enzymes, suggesting that supplementation of turmeric *exerts* protective effects against hepatocyte injury.

### 3.4 Influence of turmeric extract on apoptotic and inflammatory signals

**Figure 6:**
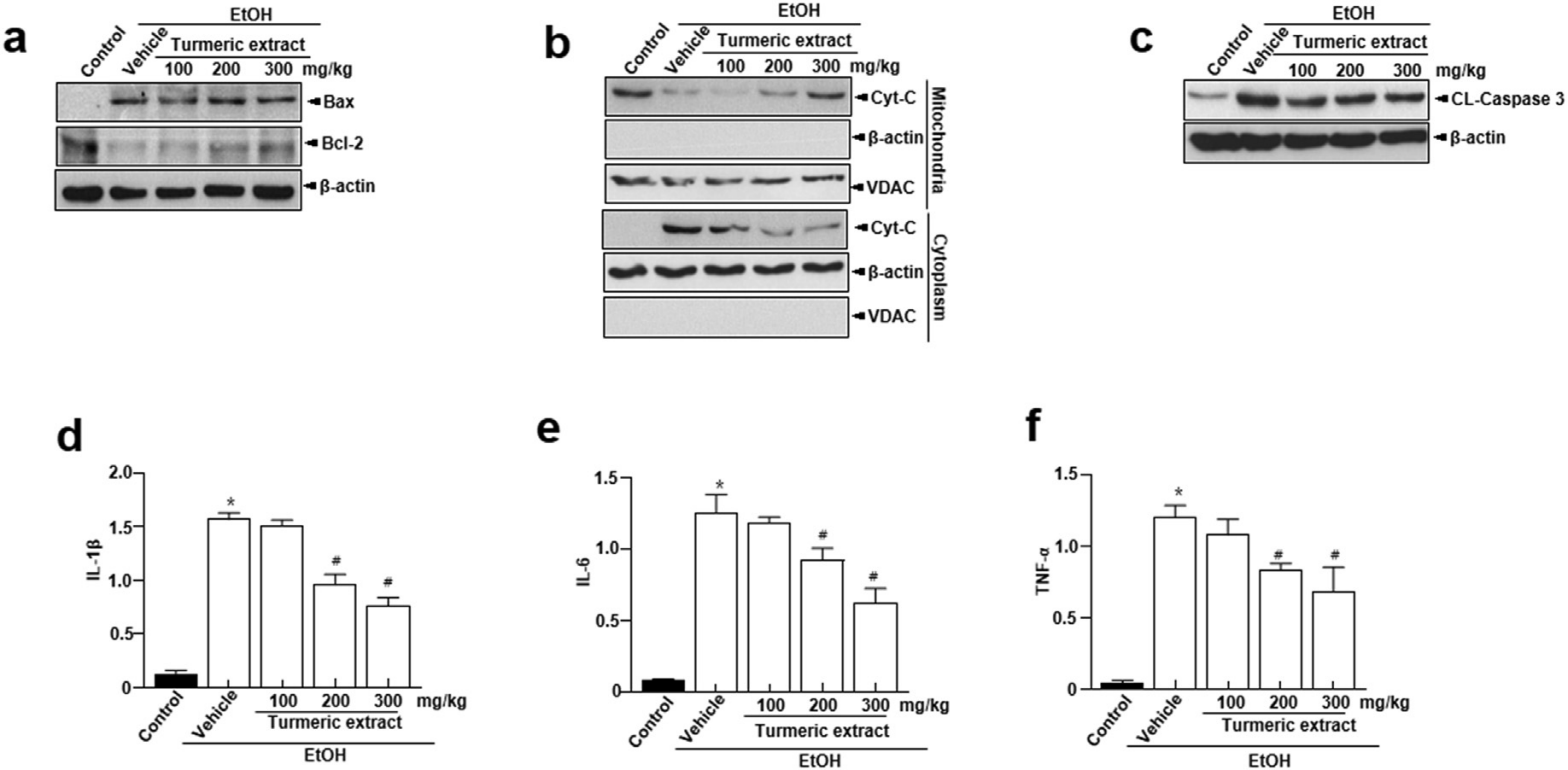
Effect of turmeric extract on the apoptotic and inflammatory signals in the liver tissue obtained from alcohol-induced SD rats. (a) Immunoblotting of Bax, Bcl-2, and β-actin. (b) Immunoblotting of Cyt C and VDAC in the mitochondria and cytoplasm. Observations depict the movement of Cyt C and VDAC. The mitochondria and cytoplasm were fractionated using liver tissue lysates. Observations confirm that VDAC is expressed only in the mitochondria. (c) Immunoblotting of the cleavage of caspase 3. (d–f) mRNA expression of inflammatory mediators (IL-1β, IL-6, and TNF-α) in the liver tissue. Data are shown as mean ± SEM (n = 8). p < 0.05 (#) represent differences from the EtOH treated group. Full and non-adjusted images of immunoblots were shown in the supplementary material (Supplementary Figure 2). Cyt C, cytochrome C; EtOH; ethanol; VDAC, voltage-dependent anion channel; IL, interleukin; TNF, tumor necrosis factor.

Apoptotic and inflammatory signals in the liver tissue are characteristic features of oxidative stress-induced liver damage (Higuchi et al., 1996). In the EtOH group, we observed increased levels of pro-apoptotic Bax protein, while the anti-apoptotic Bcl-2 protein levels were decreased (Figure 6a). Moreover, mitochondria released higher cytochrome C into the cytoplasm. However, the cytochrome C release into the cytoplasm was lower in the turmeric extract-administered groups (Figure 6b). Additionally, the EtOH group showed cleaved caspase-3, while turmeric extract pre-administration appeared to prevent cleavage (Figure 6c). Furthermore, real-time PCR observations demonstrated a substantial increase in levels of pro-inflammatory cytokines, such as IL-1β, IL-6, and TNF-α, in the EtOH group than in the turmeric extract-administered groups (Figure 6d-f).

### 3.5 The scheme of turmeric extract-induced protective mechanism against alcohol intoxicity and damage

**Figure 7:**
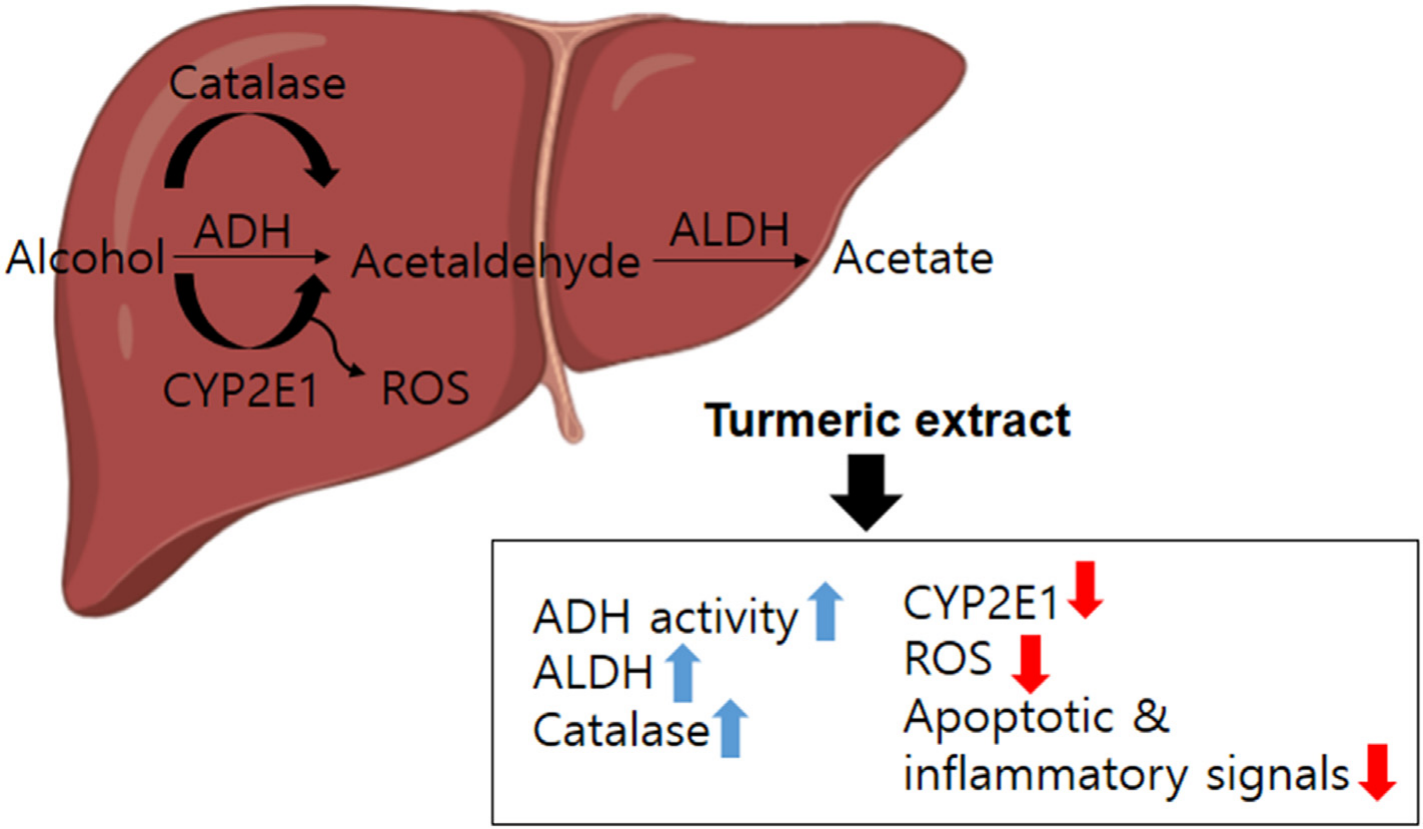
Schematic representation demonstrating the influence of turmeric extract on the liver of rodents administered with a single high dose of alcohol. Administration of turmeric extract enhances ADH, ALDH, catalase, SOD, and GPx activity, positively influencing hangover symptoms. In addition, administration of *turmeric extract* reduced ethanol-induced increase in the CYP2E1 mRNA expression and enzyme activity, ROS generation, and apoptotic and inflammatory signals.

The critically intolerable or uncomfortable hangover symptoms are associated with partially metabolized and accumulated acetaldehyde (Mackus et al., 2020). Turmeric extract improved alcohol metabolism by increasing ADH and ALDH, resulting in better alcohol clearance. Further, turmeric extract increased catalase, SOD, and GPx activity and decreased CYP2E1 expression/activity, ROS/inflammatory mediators, and apoptotic signals in EtOH-intoxicated rodents. These observations suggest that turmeric has beneficial effects against alcohol-induced hangover and its associated acute liver damage (Figure 7)*.*

## 4 Discussion

In this investigation, turmeric extract has been proved to be an effective natural substance to accelerate alcohol metabolism and enhance liver function with strong antioxidant and anti-inflammatory effects. It was observed that turmeric extract could effectively prevent liver damage in rats exposed to acute ethanol. We believe that ethanol-induced liver damage is linked to increased lipid peroxidation, formation of lipid radicals, and reduced hepatic antioxidant protection. Elevated ALT, AST, alcohols, acetaldehyde, ROS, MDA generation, and diminished ADH and ALDH activity are all indicative of ethanol-induced hepatotoxicity. The administration of turmeric extract efficiently regulated all these indicators. In addition, ethanol-induced cell death and inflammation were also prevented by turmeric extract*.* All of our findings strongly support that the supplementation of turmeric extract. Potentially reduces ethanol-induced hepatotoxicity.

Hangover symptoms typically occur in individuals when alcohol consumption exceeds 1.0 g ethanol/kg. They include both physical and mental symptoms, which may appear immediately after binge drinking or after the blood alcohol concentration drops significantly. In this study, we selected the single high dose (3 g/kg) of ethanol administration model to mimic bingeing (Figure 1). Turmeric, which has been well studied to show a protective effect against liver toxicity (Lee et al., 2016), has been applied to the rat-based acute ethanol metabolism and associated acute liver toxicity model. In this study, turmeric extract was shown to control alcohol and acetaldehyde metabolism. We observed enhanced ADH and ALDH activities upon administration of turmeric extract (Figure 3a-d), which contributed to a marked reduction in the blood acetaldehyde and ethanol concentrations (Figure 3e-f). These observations indicate that supplementation of turmeric extract enhances alcohol clearance, reducing hangover symptoms. Additionally, curcumin, a major component in turmeric extract, could help to relieve hangover symptoms (Liang et al., 2021). In humans, turmeric extract and curcumin showed lower blood acetaldehyde levels and reduced discomfort, indicating the inhibitory effects on alcohol intoxication (Liang et al., 2021; Sasaki et al., 2011). The hangover symptoms, including physical and mental symptoms, typically occur in individuals when alcohol consumption exceeds 1.0 g ethanol/kg (Piasecki et al., 2010). These symptoms may appear immediately after binge drinking or after the blood alcohol concentration drops significantly. Thus, we administered a single high dose (3 g/kg; 3.75 mL/kg) of ethanol to the experimental rats to mimic bingeing. Therefore, approximately 2.5–3 mL of 35% EtOH/rat was applied depending on the animal body weight. Safety-wise, there is a consensus that turmeric extract water and ethanol extracts are relatively safe functional food (Kocaadam and Sanlier, 2017; Liu and Nair, 2012; Mun et al., 2019), and even the high dose of 5,000 mg/kg reported no side or unexpected effect (Kim et al., 2014). Further, if we consider routinely applied doses from 30 to 500 mg/kg of turmeric extract showed functional benefits, including the hepatoprotective effect (Lee et al., 2017a; Lee et al., 2017b; Mun et al., 2019). Thus, after careful research on previous reports, we optimized the turmeric extract dose for the study.

ADH and ALDH predominantly use oxidative pathways to metabolize ethanol in the liver. These ADH and ALDH were controlled by turmeric extract, and turmeric extract treatment lowered both CYP2E1 mRNA expression and enzyme activity (Figure 4a-b). The enhanced CYP2E1 mRNA expression and activity in the EtOH group are likely responses to oxidative stress during excessive ethanol exposure (Lu and Cederbaum, 2008). Further, reduced oxidative stress observed in the liver tissue of turmeric extract-administered rat (Figure 4c-e) correlates with the reduced CYP2E1 mRNA and activity levels. In addition, catalase plays a minor role in alcohol oxidation compared to CYP2E1 or ADH, but the role of antioxidant enzymes becomes critical during bingeing. Catalase, SOD, and GPx enzyme activities were notably increased in turmeric extract-administered groups (Figure 5a-c)*.* It has been established that oxidative stress, ROS production, and an imbalance between oxidants and antioxidants are responsible for ethanol-induced liver damage (Farzaei et al., 2018; Xia et al., 2018). Among these, oxidative stress and antioxidants are crucial as they modulate several processes, including degradation of lipids and proteins, which are vital to the progression of hepatocyte injury (Farzaei et al., 2018). Primarily, increased levels of oxidants (O_2_^-^ and H_2_O_2_) and malondialdehyde (MDA) and reduced levels of antioxidants such as SOD and GSH in the liver cause oxidative stress (Lee et al., 2017c; Tang et al., 2017). Among these factors, SOD is a key antioxidant enzyme required to reduce the damage induced by oxidative stress by scavenging free radicals and accelerating their clearance (Tang et al., 2017). Similarly, GSH is another low molecular weight scavenger that protects DNA, proteins, and other cofactors from oxidative damage by removing O_2_^-^ and H_2_O_2_. Moreover, the structural integrity and function of the cell membrane are preserved by the activity of GPx by accelerating the conversion of GSH to H_2_O_2_ (Han et al., 2015). Hence, measurement of these factors is essential in determining the functional role of turmeric extract against oxidative stress. The administration of turmeric extracts efficiently regulated all these critical parameters indicative of oxidative stress, suggesting the significant influence of turmeric extract against oxidative stress. The study observations reveal that turmeric extract prevents ethanol-induced hepatic injury and inflammations in alcohol-intoxicated rats by modulating the expression of Bcl-2, Bax, cleaved caspase-3, and the translocation of cytochrome C, a classical apoptosis signaling process signaling (Figure 6a-c) and induction of pro-inflammatory cytokines such as IL-1β, IL-6, and TNF-α (Figure 6d-f). The antioxidant benefits of turmeric extract and its major component, curcumin, have been most widely studied in the literature (Abrahams et al., 2019; Lin et al., 2019; Menon and Sudheer, 2007). Curcumin potentially contributes to its antioxidant activity via scavenging ROS, H_2_O_2_, NO and by suppressing lipid peroxidation (Ak and Gulcin, 2008). These actions could be attributed to enhanced antioxidant enzyme activities, including SOD, catalase, GPx, and heme oxygenase-1 (HO-1). Curcumin could increase the GSH levels by upregulating glutathione transferase and their mRNAs, which indicates the ability of turmeric extract and curcumin to prevent hepatic injury and inflammation via enhanced alcohol clearance and modulating antioxidants.

In summary, the administration of turmeric extract potentially preserves the optimal levels of key modulators and restores binge drinking-induced liver damage by suppressing lipid peroxidation, oxidative stress, and enhancing antioxidant defense. This study also offers turmeric extract as an alternative natural functional food product to prevent the negative implications of binge drinking.

## Ethics statement

All the experimental animals used in this study were cared for in accordance with the regulations of the Care and Use of Laboratory Animals Guide of Jeonbuk National University with approval from the Institutional Animal Care and Use Committee of Jeonbuk National University Hospital (cuh-IACUC-2019-09).

## Declarations

### Author contribution statement

Hwa-Young Lee and Geum-Hwa Lee: Conceived and designed the experiments; Performed the experiments; Analyzed and interpreted the data; Wrote the paper.

The-Hiep Hoang, Seung Wook Kim, Choon Gil Kang and Jae Hyeok Jo: Performed the experiments.

Myoung Ja Chung, Kyunghyun Min and Han-Jung Chae: Interpreted the data; Wrote the paper.

### Funding statement

This work was supported by Ottogi Corporation.

### Data availability statement

Data will be made available on request.

### Declaration of interest’s statement

The authors declare no conflict of interest.

### Additional information

No additional information is available for this paper.

## Appendix A Supplementary data

supple material.pdf supple material.pdf
