# Supplementary material for: Turmeric extract (Curcuma longa L.) regulates hepatic toxicity in a single ethanol binge rat model
Source: Heliyon. 2022 Sep 22;8(9):e10737. doi: 10.1016/j.heliyon.2022.e10737 (PMC9526153; doi:10.1016/j.heliyon.2022.e10737)

Supplementary Figure 1. Ethanol-induced CYP2E1 activity and oxidative stress is suppressed by turmeric extract. (Figure 4a)

Figure 4a

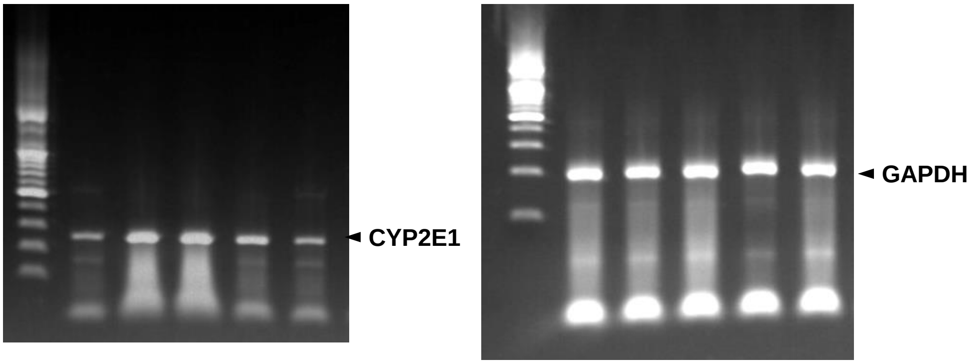

Supplementary Figure 2. Influence of turmeric extract on the activity of antioxidant enzymes in alcohol-induced SD rats. (Figure 6a, Figure 6b, and Figure 6c)

Figure 6a

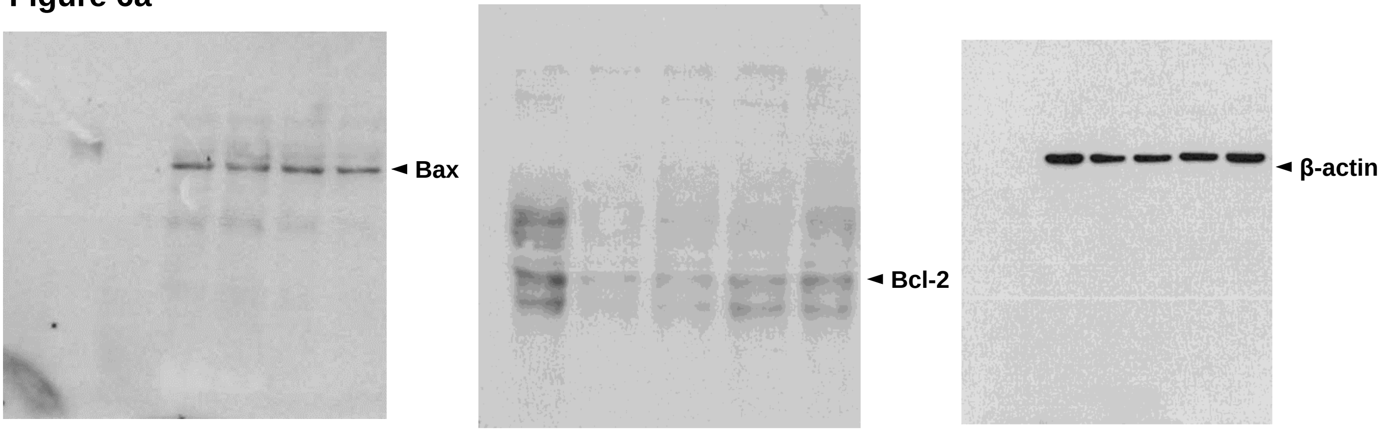

Figure 6b

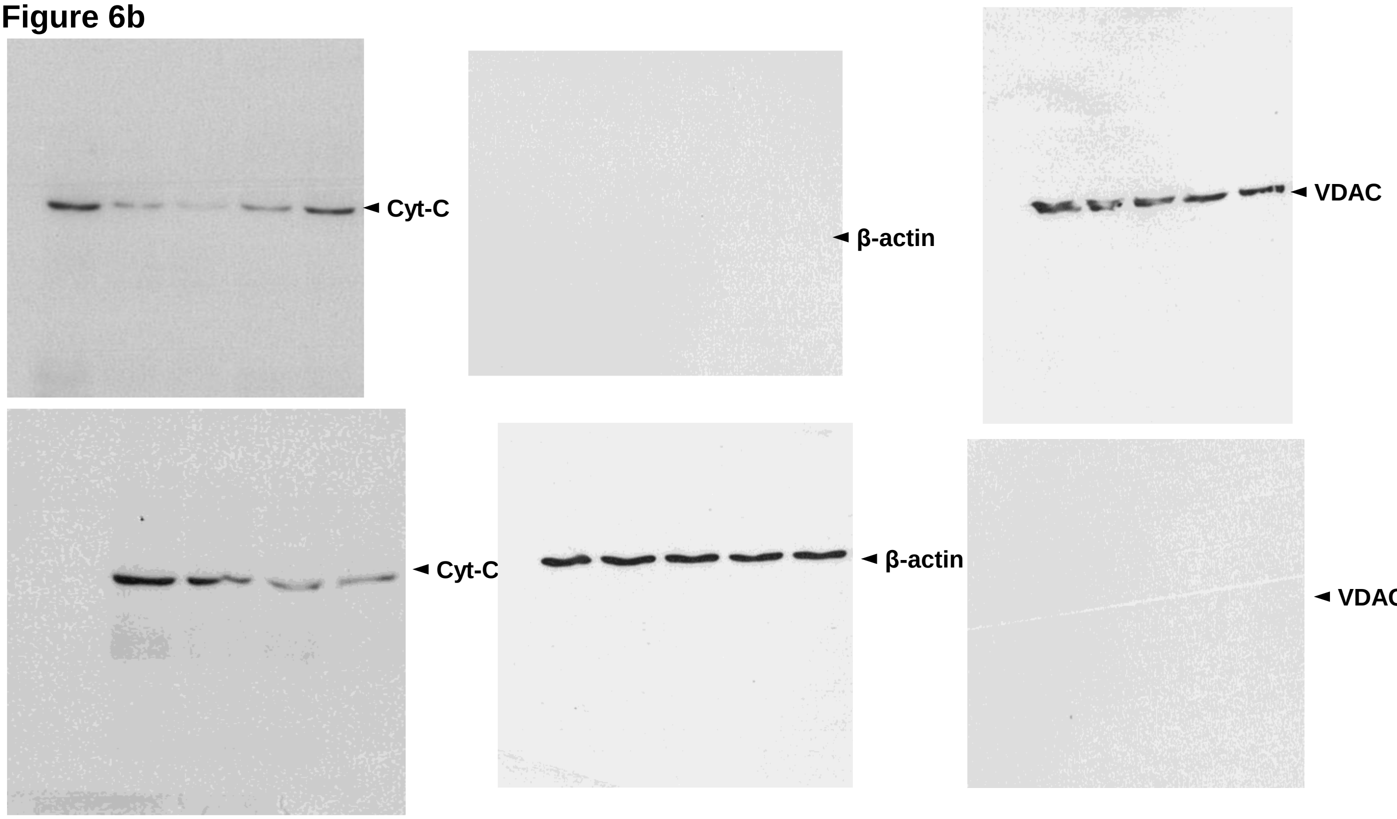

Figure 6c

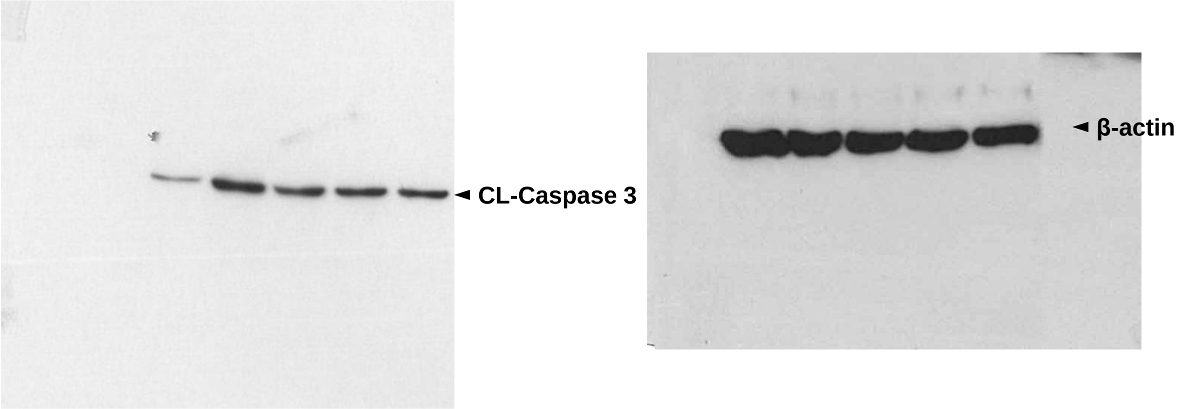

Supplement: supple material.pdf [file mmc1.pdf]
